# The impact of waist circumference on function and physical activity in older adults: longitudinal observational data from the osteoarthritis initiative

**DOI:** 10.1186/1475-2891-13-81

**Published:** 2014-08-09

**Authors:** John A Batsis, Alicia J Zbehlik, Laura K Barre, Todd A Mackenzie, Stephen J Bartels

**Affiliations:** Section of General Internal Medicine, Dartmouth-Hitchcock Medical Center, 1 Medical Center Drive, Lebanon, NH 03756 USA; Centers for Health and Aging, 46 Centerra Parkway, Lebanon, NH 03766 USA; Geisel School of Medicine at Dartmouth, 1 Rope Ferry Road, Hanover, NH 03755 USA; Section of Rheumatology, Dartmouth-Hitchcock Medical Center, 1 Medical Center Drive, Lebanon, NH 03756 USA; The Dartmouth Institute, Dartmouth College, 30 Lafayette St, Lebanon, NH 03766 USA; Division of Nutritional Sciences, School of Human Ecology, Cornell University, Ithaca, NY 14850 USA

**Keywords:** Obesity, Waist circumference, Osteoarthritis, Disability, Physical activity, Quality of life

## Abstract

**Background:**

We previously demonstrated that BMI is associated with functional decline and reduced quality of life. While BMI in older adults is fraught with challenges, waist circumference (WC) is a marker of visceral adiposity that can also predict mortality. However, its association with function and quality of life in older adults is not well understood and hence we sought to examine the impact of WC on six-year outcomes.

**Methods:**

We identified adults aged ≥60 years from the longitudinal Osteoarthritis Initiative and stratified the cohort into quartiles based on WC. Our primary outcome measures of function at six year follow-up included: self-reported quality of life [Short Form-12 (SF-12)], physical function [Physical Activity Scale for the Elderly (PASE)] and disability [Late-life Disability Index (LLDI)]. Linear regression analyses predicted 6-year outcomes based on WC quartile category (lowest = referent), adjusted for age, sex, race, education, knee pain, smoking status, a modified Charlson co-morbidity index and baseline scores, where available.

**Results:**

We identified 2,182 subjects meeting our inclusion criteria and stratified the study cohort by quartiles of WC. Mean age ranged from 67.5-68.7 years, 60-71% were female and 80-86% were white. The highest WC quartile compared to 50-75th, 25-50th or lowest quartile, was associated with a greater number of medications (4.3, 4.0, 3.6 and 3.4 [p < 0.001]), lower gait speeds (1.23, 1.27, 1.32, and 1.34 m/s[p < 0.001]), higher rates of knee osteoarthritis (70.2, 62.2, 60.2, 48.6;p < 0.001), higher Charlson co-morbidity scores and greater knee pain (WOMAC scores) (all p < 0.001). At follow-up, adjusted SF-12 physical function subscale and PASE scores, were lowest in the highest WC quartile as compared to the 50-75%, 25-50%, and lowest quartiles [(SF-12 scores: 45.5, 46.7, 47.6, and 47.9), and (PASE scores: 109.6, 128.7, 126.6, and 131.0). The LLDI limitation subscale for disability demonstrated lower scores in the high WC quartile as opposed to the referent group.

**Conclusions:**

Elevated WC is associated with lower quality of life, a decline in physical function, and a slightly higher risk of disability over time. Intervention studies are needed to prevent functional decline in this high-risk population.

## Background

A number of physiological alterations occur during the aging process leading to impairments in activities of daily living that can lead to disability, institutionalization and death [1]. Osteoarthritis (OA) is an increasingly prevalent cause of functional decline and can augment the changes in body composition that occur with aging [2, 3]. The observed decline in mobility subsequently places older adults at increased risk of falls and dependency.

Along with the rising number of older adults, the prevalence of obesity measured by body mass index continues to rise. A recent population-based survey in the United States found that 35% of older adults are obese [4], paralleling the prevalence observed in the general population. Adiposity is associated with increased cardiometabolic risk [5], and in older adults can lead to diminished quality of life and disability [6, 7]. Yet, BMI-defined obesity has poor sensitivity and specificity in identifying adiposity [8], and cannot distinguish between subcutaneous and visceral adiposity.

Waist circumference (WC) has been proposed as a relatively inexpensive measure of visceral adiposity. An elevated WC is one of five criteria that define the metabolic syndrome [9], which itself has been associated with functional decline, frailty and disability [10]. Cross-sectional and longitudinal studies suggest an association of increased BMI with mobility impairments [11]. However, to our knowledge, there have been few longitudinal studies of older adults examining whether WC as a surrogate marker for visceral adiposity is associated with distal geriatric outcomes, including falls, functional decline and quality of life [11, 12].

Obesity is a major risk factor for increasing severity of early degenerative changes and cartilaginous lesion progression of knee OA [13], placing older patients at risk for worsened musculoskeletal disorders and functional decline [14, 15]. We previously demonstrated in this cohort that obesity, measured using a body mass index (BMI) ≥30 kg/m^2^, is associated with reduced quality of life, reduced physical activity and increased risk of disability [Batsis Submitted *Public Health Nutrition*] Examining the impact of central adiposity in this high-risk population allows clinicians not only to determine whether WC is a harbinger of disability but also whether it can be used as an alternative anthropometric measure. We hypothesize that in a cohort of older adults at risk for OA, that long-term quality of life, physical activity and activities of daily living are compromised in older adults with elevated WC.

## Methods

The Osteoarthritis Initiative (OAI) is a multi-center, longitudinal, prospective observational study of osteoarthritis in adults. Data collection began in 2004 and is gathered at four clinical sites: Baltimore, MD, Pawtuckett, RI, Pittsburgh, PA, and Columbus, OH. The study is funded by the National Institute of Arthritis, Musculoskeletal and Skin Diseases.

Subjects were recruited through mailings, advertisements, and community meetings. To ascertain subject eligibility, a telephone interview was conducted, and if eligible, subjects attended a screening clinic visit where additional assessments were performed. Baseline data including questionnaires, interviews, and physical assessments were collected at an enrollment clinic visit. All such visits occurred within a six-week period. Inclusion criteria included recruiting equal numbers of males and females, aged 45–79 years, and all ethnic groups were eligible for the study. Patients were excluded if they had rheumatoid arthritis, severe joint space narrowing; bilateral total knee replacements; inability to undergo an MRI; unable to provide blood samples; comorbidity preventing study participation; unlikely to be resident in the area for at least 3 years; other research participation; and unwillingness to sign an informed consent. Protocols are available online for full review. All data is freely available for download at http://www.oai.ucsf.edu/. Participants completed follow up assessments at yearly intervals and most recent study follow-up consists of six year data. For this study, baseline and six-year outcome data was used. Our local institutional review board, the Committee for the Protection of Human Subjects (CPHS#23707), deemed the study exempt for research purposes.

### Study population

The OAI study sample consisted of subjects of both sexes and of all ethnicities aged 45–79 years. All subjects were classified at baseline according to one of three subgroups: clinically significant knee osteoarthritis at risk of disease progression (progression); subjects at high risk of developing clinically significant knee OA (incident); and a control group. The progression subgroup patients complained of frequent knee symptoms or radiographic tibiofemoral knee OA in at least one native knee. The incident cohort was free of baseline symptomatic knee OA, but had established risk factors including the presence of heberden’s nodes in both hands, increased weight; previous knee operation; previous knee injury; family history; and pain in the knee on most days of the preceding month. The control group did not have pain nor radiographic findings or risk factors.

We identified 2,585 subjects aged ≥ 60 years and excluded subjects with missing measures of adiposity (body mass index [BMI] (n = 1) or waist circumference [WC] (n = 38)); BMI <18.5 kg/m^2^ (n = 4), and those dead at follow-up (n = 166). We excluded subjects without baseline primary outcome measures (short-form 12 (n = 21) or physical activity scores (n = 15)). Subjects with incident total knee arthroplasty (n = 158) were also excluded to allow the ascertainment of the natural history of osteoarthritis in the absence of surgical intervention for the knee. The final cohort consisted of 2,182 subjects.

### Study measures

All study-related data was obtained by patient self-report or by measurements based on study protocols. Questionnaires collected data on baseline demographic, medical, social and ethnic characteristics of subjects. Age at the initial visit was considered age at baseline. We dichotomized marital status as ‘married’ or ‘single’, with the latter consisting of widow, divorced, separated or never married. Education status was classified as follows: high school (graduated or not); attended college; college graduate; graduate level. Ever smokers were considered patients who smoked >100 cigarettes in their life. The Western Ontario and McMaster University OA Index (WOMAC) Pain Scale was utilized to assess self-reported knee pain on a 5-point Likert scale. Pain scores ranged from 0–20 with higher scores representing worse symptoms. Subjects with knee osteoarthritis on x-ray were considered to have knee OA. The Charlson co-morbidity index captured the degree of medical co-morbidity in the cohort. Subjects were classified as having an impairment in an activity of daily living if they were unable to perform any of the following: dressing, walking, bathing, eating, getting out of bed, or toileting.

### Measurements

Participant weight was measured without shoes or heavy jewelry, wearing light clothing, using a calibrated standard balance beam scale. Standing height was measured with a wall-mounted stadiometer. Waist circumference was measured at the level of the umbilicus between the lower rib and the iliac crest. BMI was calculated as weight (in kilograms) divided by height (in meters) squared. Gait speed was measured using the 20 m walk test, a validated measure of functional status in people with knee osteoarthritis [16]. Participants walked 20 m in an unobstructed corridor at their usual walking speed and were timed. Reliability and intra-class correlations ranged from 0.93 to 0.98 within and between testers in patients with moderate to severe knee OA.

### Outcome measures

The Short-Form 12 (SF12) is a self-reported valid and reliable measure of a person’s perceived health status [17]. The overall score accounts for >90% of the statistical variance of the longer Short Form 36 scale [18], and is valid, reliable and easily self-administered Physical and mental component scores are assessed on a Likert scale, and overall SF-12 score combines the two assessments. Overall scores range from 0 to 100 and was administered as a take-home questionnaire.

The Physical Activity Scale for the Elderly (PASE) [19] is a measure of occupational, household, and leisure activities during a one-week period in older adults >65 years. This 26-item instrument is reliable and valid, and can be administered by telephone, mail or be administered in-person. In this study, the scale was administered in person. The general population has a mean score of 103 ± 64.1, with greater scores indicating greater intensity of activity. However, no known minimally clinically important differences are available.

The validated Late-Life Function and Disability Instrument (LLDI) [20] focuses on functional limitations and frequency limitations. These domains parallel Nagi’s disablement framework [21] on disability in community-dwelling adults. Functional limitations reflect a person’s inability to perform daily activities, while frequency limitations characterize the inability to engage in social environments and major life tasks. Higher scores indicate higher levels of function and is scored on a 0–100 scale. The scale corresponds to both the physical functioning subscale of the SF-36 and London Handicap Scale [22].

### Statistical analysis

All data are represented as either means ± standard deviation, or counts (percent) (Table 1). The cohort was stratified by waist circumference quartiles [<95.1, 95.1-103.2, 103.2-111, 111–149.3 cm] Baseline characteristics across groups were compared using chi square and one-way ANOVA tests. We used paired t-tests to compare baseline to six year follow-up data (Table 2). To determine the association of baseline WC on functional outcomes at six years we used multiple linear regression. The primary six-year outcomes of interest included SF-12, PASE score, and LLDI scoring. Our primary predictor was WC using quartiles (referent = lowest quartile). As differences may be introduced simply because categories reflect different points in the distribution, researchers often control for this effect by dividing variables into categories of equal size [23]. Three separate regression analyses were performed. We determined the mean follow-up scores for each functional outcome in each of the WC categories after adjusting for age, sex, education level, race, cohort type (incident, progression and control), Charlson co-morbidity score [24], current smoking status, and adjusting for baseline scores where available (Tables 3 and 4). As many indicator variables are associated with both obesity and our outcome measures, we adjusted in our analysis for a modified Charlson index including chronic obstructive pulmonary disease, ulcers, renal disorders, kidney transplant, rheumatoid arthritis, and cancer Tables 5 and 6. Other variables excluded from this composite score included subjects that had not fulfilled criteria for these at baseline. Second, we stratified our cohort by age, as disability is known to change with advanced age. Separate analyses were performed on the 60–70 year age group and the ≥ 70 year age group. Third, we stratified our analyses by knee osteoarthritis (present/absent). We performed inverse probability weighting to account for missing outcome data at follow-up. Adjusted means (95% confidence intervals), or mean differences compared to the lowest WC quartile are presented. Body weight and body mass index were highly collinear and hence were not included in our modeling. We present the R^2^ of models using standard BMI categories and WC quartiles in Table 7. All estimates were calculated using STATA version 12 (STATACorp, College Station, TX). A p value of <0.05 was considered statistically significant.

**Table 1:** **Baseline Characteristics of all subjects >60 years old (n = 2,182)**

			Waist circumference	Quartile		
	Overall cohort	Low	25-50%	50-75%	75%+	p-value
	N = 2,182	n = 553	n = 538	n = 548	n = 543	
**Age, years**	68.2 ± 5.4	67.9 ± 5.4	68.7 ± 5.4	68.5 ± 5.4	67.5 ± 5.3	<0.001
**Female Sex, %**	1367 (62.7)	390 (70.5)	326 (60.6)	327 (59.7)	324 (59.7)	<0.001
**Education Status**						
**< High School**	432 (19.8)	93 ( 16.8)	111 (20.6)	119 (21.7)	109 (20.1)	
**Some College**	524 (24.0)	125 (22.6)	134 (24.9)	128 (23.4)	137 (25.2)	0.23
**College**	417 (19.1)	103 (18.6)	101 (18.7)	99 (18.1)	114 (21.0	
**>College**	809 (37.1)	232 (42.0)	192 (35.7)	202 (36.9)	183 (33.7)	
**Yearly Income**						
**>$50,000**	1115 (53.7)	301 (57.0)	280 (55.2)	285 (54.7)	249 (47.8)	0.02
**Marital Status**						
**Married**	1443 (66.2)	370 (67.0)	368 (68.4)	376 (68.6)	329 (60.6)	0.02
**Race**						
**White**	1792 (82.2 )	473 (85.7)	434 (80.7)	451 (82.3)	434 (79.9)	
**Black**	336 (15.4)	62 (11.2)	90 (16.7)	85 (15.5)	99 (18.2)	0.003
**Asian**	53 (2.4)	9 (1.6)	8 (1.5)	2 (0.4)	--	
**Charlson Score**	0.45 ± 0.9	0.31 ± 0.73	0.45 ± 0.89	0.48 ± 0.96	0.58 ± 0.99	<0.001
**Modified Charlson Score**	0.09 ± 0.30	0.05 ± 0.21	0.09 ± 0.31	0.12 ± 0.23	0.12 ± 0.34	<0.001
**WOMAC pain Right**	16.0 ± 15.5	8.4 ± 11.9	10.8 ± 13.4	12.3 ± 14.3	14.2 ± 15.3	<0.001
**WOMAC pain Left**	16.4 ± 16.8	7.8 ± 12.6	10.8 ± 14.6	12.0 ± 15.3	13.8 ± 16.1	<0.001
**Ever Smoker**	1084 (50.1)	258 (46.8)	256 (48.1)	289 (53.1)	281 (52.2)	0.10
**# Medications**	3.83 ± 2.44	3.41 ± 2.27	3.61 ± 2.30	3.99 ± 2.49	4.27 ± 2.59	<0.001
**Knee Osteoarthritis Present**	1315 (60.3)	269 (48.6)	324 (60.2)	341 (62.2)	381 (70.2)	<0.001
**Waist circumference, cm**	103.2 ± 12.1	88.4 ± 5.9	98.9 ± 2.2	106.7 ± 2.3	118.7 ± 6.8	<0.001
**Body Mass Index, kg/m** ^**2**^	28.3 ± 4.5	24.1 ± 2.9	26.9 ± 3.0	29.2 ± 2.9	33.2 ± 3.4	<0.001
**Cohort Allocation**						
**Incident**	1,595 (73.1)	453 (81.9)	401 (74.5)	397 (72.5)	344 (63.4)	
**Progression**	577 (26.4)	96 (17.4)	133 (24.7)	150 (27.4)	198 (36.5)	<0.001
**Control**	10 (0.5)	4 (0.7)	4 (0.7)	1 (0.2)	1 (0.2)	

All values represent mean ± SD, or count (%).

P-value represents the ANOVA across all waist circumference quartiles categories.

*Abbreviations*: OA defined as having radiographic knee osteoarthritis on either knee or both knees, WOMAC Western Ontario and McMaster University Arthritis Index.

Some fields may not add up to overall cohort totals due to missing values.

Modified Charlson score consisted of the following variables: chronic obstructive pulmonary disease, ulcers, renal disorders, kidney transplant, rheumatoid arthritis, and cancer.

**Table 2:** **Primary and follow-up outcome measures – unadjusted quartiles (n = 2,182)**

			Waist circumference	Quartile	e	
	Overall cohort	low	25-50%	50-75%	75%+	p-value
**SF-12 Score**						
**Total**						
**Baseline**	103.5 ± 11.1	106.0 ± 9.9	104.3 ± 10.7	102.3 ± 11.6	101.2 ± 11.3	<0.001
**Follow-up**	101.1 ± 12.4	103.9 ± 11.3	102.4 ± 12.3	99.4 ± 12.7	98.5 ± 12.7	<0.001
**p-value**	<0.001	<0.001	<0.001	<0.001	<0.001	
**Physical**						
**Baseline**	48.7 ± 8.9	51.3 ± 7.9	49.4 ± 8.7	47.7 ± 9.3	46.5 ± 9.11	<0.001
**Follow-up**	46.4 ± 9.7	49.0 ± 8.8	47.4 ± 9.7	45.0 ± 9.7	44.0 ± 10.0	<0.001
**p-value**	<0.001	<0.001	<0.001	<0.001	<0.001	
**Mental**						
**Baseline**	54.8 ± 7.5	54.8	54.9	54.6	54.7	0.90
**Follow-up**	54.7 ± 8.1	54.9 ± 7.5	55.0 ± 7.5	54.4 ± 8.6	54.5 ± 8.9	0.63
**p-value**	0.21	0.71	0.89	0.43	0.23	
**PASE**						
**Baseline**	136.4 ± 67.2	146.9 ± 68.3	139.4 ± 68.2	130.7 ± 67.0	128.8 ± 63.8	<0.001
**Follow-up**	123.7 ± 64.7	133.1 ± 64.2	125.3 ± 63.5	126.8 ± 68.2	108.4 ± 58.7	<0.001
**p-value**	<0.001	<.001	<0.001	0.01	<0.001	
**LLDI**						
**Frequency**	55.3 ± 6.3	56.2 ± 6.3	54.9 ± 6.1	55.0 ± 6.4	54.9 ± 6.3	0.004
**Limitation**	80.9 ± 15.3	83.6 ± 15.0	82.3 ± 15.2	79.4 ± 15.2	78.3 ± 15.2	<0.001
**ADL Impairment**	172 (10.6)	31 (7.3)	36 (8.9)	42 (10.4)	63 (16.6)	<0.001
**Gait Speed**						
**Baseline**	1.29 ± 0.21	1.34 ± 0.22	1.32 ± 0.22	1.27 ± 0.20	1.23 ± 0.20	<0.001
**Follow-up**	1.24 ± 0.21	1.30 ± 0.21	1.25 ± 0.22	1.23 ± 0.19	1.16 ± 0.21	<0.001
**p-value**	<0.001	<0.001	<0.001	<0.001	<0.001	

All values represent mean ± SD, or count (%).

P-value represents the difference between the excluded and included analytical cohort.

*Abbreviations*: ADL Activities of Daily Living, LLDI Late-life disability index, PASE Physical Activity Scale for the Elderly, SF- Short Form.

**Table 3:** **Multivariable regression analysis of follow-up primary outcome measures (n = 2,182)**

		Waist	Circumference	Quartile	
		Low WC	25-50%ile	50-75%ile	75%+
**Short-Form 12 Score**					
**Overall Score**	**Overall**	102.4 [101.0-103.9]	102.7 [101.5-103.9]	101.7 [100.7-102.8]	100.5 [99.2-101.8]
	**Δ**	---	0.28	-0.69	-1.92 (p = 0.04)
	**60-70**	102.9 [101.0-104.9]	103.0 [101.3-104.8]	102.5 [101.2-103.9]	101.4 [99.8-103.1]
	**Δ**	---	0.10	-0.46	-1.50
	**70+**	101.5 [99.0-104.0	102.1 [100.3-104.0]	100.3 [98.5-102.1]	99.1 [96.9-101.2]
	**Δ**	---	0.62	-1.22	-2.45
**Physical**	**Overall**	47.9 [46.9-48.8]	47.6 [46.7-48.5]	46.7 [45.8-47.5]	45.5 [44.5-46.0]
	**Δ**	---	-0.26	-1.20	-2.3 (p = 0.001)
	**60-70**	47.8 [46.6-49.1]	48.7 [47.5-49.8]	47.0 [45.8-48.2]	46.2 [44.9-47.6]
	**Δ**	---	0.83	-0.85	-1.61
	**70+**	47.7 [46.0-49.4]	46.4 [45.0-47.9]	46.2 [44.9-47.5]	44.4 [42.9-46.0]
	**Δ**	---	-1.26	-1.54	-3.26 (p = 0.004)
**Mental**	**Overall**	55.2 (54.4-55.9)	55.3 (54.5-56.1)	54.9 (54.2-55.7)	54.7 (53.8-55.5)
	**Δ**	---	-0.39	0.16	-0.40
	**60-70**	55.3 (54.3-56.2)	54.9 (53.8-55.9)	55.4 (54.5-56.4)	54.9 (53.8-56.0)
	**Δ**	---	-0.39	0.16	-0.40
	**70+**	54.9 (53.6-56.2)	55.6 (54.4-56.8)	54.3 (53.0-55.0)	54.5 (53.1-55.9)
	**Δ**	---	0.71	-0.60	-0.37
**Physical Activity Scale for Elderly**	**Overall**	131.0 (124.3-137.7)	126.6 (120.3-132.8)	128.7 (121.5-135.9)	109.6 (102.6-116.6)
	**Δ**	---	-4.47	-2.34	-21.4 (p < 0.001)
	**60-70**	137.4 (127.8-146.9)	136.4 (12.7-145.1)	136.4 (126.7-146.2)	119.9 (111.1-128.7)
	**Δ**	---	-1.0	-0.97	-17.5 (p = 0.006)
	**70+**	117.5 (109.5-125.5)	112.2 (103.4-121.0)	114.6 (105.3-124.0)	93.5 (82.8-104.0)
	**Δ**	---	-5.2	-2.83	-23.9 (p < 0.001)
**Late-Life Disability Index**					
**Frequency**	**Overall**	56.0 (55.3-56.7)	55.6 (54.9-56.2)	55.9 (55.2-56.5)	55.1 (54.4-55.8)
	**Δ**	---	-0.43	-0.17	-0.94
	**60-70**	56.7 (55.8-57.6)	56.1 (55.1-57.0)	56.1 (55.2-57.8)	55.7 (54.8-56.6)
	**Δ**	---	-0.66	-0.61	-1.1
	**70+**	55.0 (54.0-56.0)	54.8 (53.9-55.8)	55.4 (54.4-56.5)	54.1 (53.0-55.2)
	**Δ**	--	-0.20	0.43	-0.90
**Limitation**	**Overall**	83.3 (81.6-84.9)	83.2 (81.6-84.8)	81.8 (80.3-83.3)	79.2 (77.5-81.0)
	**Δ**	--	-0.07	-1.47	-4.05 (p = 0.001)
	**60-70**	84.2 (82.1-86.4)	84.6 (82.4-86.8)	83.2 (81.2-85.1)	80.9 (78.9-82.9)
	**Δ**	--	0.38	-1.06	-3.3 (p = 0.026)
	**70+**	81.8 (79.3-84.4)	81.1 (78.6-83.5)	80.0 (77.6-82.4)	76.3 (73.1-79.4)
	**Δ**	---	-0.77	-1.86	-5.56 (p = 0.006)
**Gait Speed**	**Overall**	1.28 (1.26-1.31)	1.25 (1.23-1.27)	1.25 (1.23-1.26)	1.19 (1.17-1.22)
	**Δ**	---	-0.03 (p = 0.018)	-0.04 (p = 0.06)	-0.09 (p < 0.001)
	**60-70**	1.29 (1.26-1.32)	1.28 (1.25-1.31)	1.27 (1.24-1.29)	1.24 (1.21-1.27)
	**Δ**	---	-0.02	-0.02	0.05 (p = 0.009)
	**70+**	1.27 (1.23-1.31)	1.19 (1.15-1.23)	1.21 (1.17-1.24)	1.11 (1.07-1.16)
	**Δ**	--	-0.08 (p = 0.002)	-0.06 (p = 0.015)	-0.15 (p < 0.001)

Values represent mean score (95% confidence interval) of the indicated metric adjusted for: Age, sex, education level, race, modified Charlson co-morbidity index, smoking status, knee OA, hip pain and cohort type (incident, progression or control), and baseline scoring (SF-12, PASE) where available. Values in 25-50%, 50-75%, high WC quartile columns represent difference from referent category (low WC). Models stratified by age adjust for the similar co-variates other than age.

Modified Charlson score consists of the following variables: chronic obstructive pulmonary disease, ulcers, renal disorders, kidney transplant, rheumatoid arthritis, and cancer.

**Table 4:** **Multivariable regression analysis of follow-up primary outcome measures (n = 2182)**

		Waist	Circumference	Quartile	
		Low WC	25-50% WC	50-75% WC	75%+
**Short-Form 12 Score**					
**Overall Score**	**OA +**	100.1 [97.6-102.6]	101.2 [99.6-102.9]	100.4 [99.0-101.7]	99.9 [98.3-101.4]
	**Δ**	---	1.18	0.32	-0.19
	**OA -**	105.6 [104.4-106.9]	105.0 [103.3-106.7]	104.3 [102.6-105.9]	101.9 [99.7-104.0]
	**Δ**	---	-0.63	-1.37	-3.77 (p = 0.002)
**Physical**	**OA +**	47.0 [45.7-48.2]	46.2 [45.1-47.4]	45.1 [43.9-46.2]	44.5 [43.2-45.7]
	**Δ**	---	-0.71	-1.90 (p = 0.02)	-2.50 (p = 0.003)
	**OA -**	50.2 [49.0-51.4]	49.9 [48.4-51.3]	49.8 [48.6-51.0]	47.6 [45.9-49.3]
	**Δ**	---	-0.33	-0.38	-2.60
**Mental**	**OA +**	54.9 [53.9-55.9]	55.1 [54.0-56.2]	55.2 [54.2-56.3]	55.5 [54.5-56.5]
	**Δ**	---	0.16	0.33	0.57
	**OA -**	55.3 [54.3-56.4]	55.4 [54.4-56.5]	54.4 [53.2-55.5]	53.0 [51.4-54.6]
	**Δ**	---	0.09	-0.93	-2.21 (p = 0.015)
**Physical Activity Scale for Elderly**	**OA +**	128.2 [119.3-137.2]	121.0 [113.7-128.3]	128.5 [119.7-137.4]	107.8 [99.6-116.0]
	**Δ**	---	-7.3	0.31	-20.4 (p < 0.001)
	**OA -**	137.5 [128.1-146.9]	136.5 [125.1-147.9]	129.8 [117.7-141.9]	111.7 [99.6-123.8]
	**Δ**	---	-1.0	-7.7	-25.8 (p = 0.001)
**Late-Life Disability Index**					
**Frequency**	**OA +**	55.7 [54.8-56.6]	55.4 [54.6-56.3]	55.1 [54.4-55.9]	54.9 [54.1-55.7]
	**Δ**	---	-0.28	-0.59	-0.84
	**OA -**	56.8 [55.9-57.7]	55.7 [54.6-56.8]	57.2 [55.9-58.4]	55.4 [54.1-56.8]
	**Δ**	---	-1.10	0.36	-1.38
**Limitation**	**OA +**	82.0 [79.9-84.2]	81.8 [79.7-83.9]	81.3 [79.4-83.1]	79.0 [76.9-81.0]
	**Δ**	---	-0.18	-2.37	-6.12 (p = 0.002)
	**OA -**	85.6 [83.6-87.7]	85.5 [82.9-88.0]	83.3 [80.8-85.8]	79.5 [76.2-82.9]
	**Δ**	---	-0.22	-0.79	-3.08 (p = 0.04)
**Gait Speed**	**OA +**	1.27 [1.24-1.30]	1.23 [1.20-1.26]	1.23 [1.21-1.25]	1.18 [1.15-1.21]
	**Δ**	---	-0.04 (p = 0.04)	-0.04 (p = 0.017)	-0.09 (p < 0.01)
	**OA -**	1.32 [1.29-1.34]	1.28 [1.25-1.31]	1.28 [1.24-1.31]	1.21 [1.17-1.25]
	**Δ**	---	-0.03	-0.036	-0.11 (p < 0.001)

**Δ** – represents the adjusted difference between each tertile of Waist Circumference (WC) and low WC.

Models were adjusted for age, sex, race, education, Charlson co-morbidity index, smoking status, and cohort type. Baseline scores were included where available.

Modified Charlson score consisted of the following variables: chronic obstructive pulmonary disease, ulcers, renal disorders, kidney transplant, rheumatoid arthritis, and cancer.

**Table 5:** **Multivariable Regression Analysis of Follow-up Primary Outcome Measures (n=2,182) – Using Charlson Co-Morbidity Index as a Co-Variate**

		Waist	Circumference	Quartile	
		Low WC	25-50%ile	50-75%ile	75%+
**Short-Form 12 Score**					
**Overall Score**	**Overall**	100.4 [98.3-102.5]	101.8 [100.7-102.8]	100.4 [99.4-101.4]	99.9 [98.8-101.0]
	**Δ**	---	1.39	-0.02	-0.53
	**60-70**	99.5 [95.1-103.8]	102.4 [101.0-103.9]	101.5 [100.2-102.7]	100.9 [99.6-102.2]
	**Δ**	--	2.96	2.00	1.43
	**70+**	100.7 [98.5-102.8]	100.8 [99.2-102.3]	98.5 [96.8-100.2]	98.2 [96.3-100.0]
	**Δ**	--	0.08	-2.18	-2.53 (p=0.05)
**Physical**	**Overall**	46.8 [45.5-48.0]	46.8 [46.0-47.6]	45.6 [44.8-46.5]	45.2 [44.3-46.0]
	**Δ**	---	0.02	-1.13	-1.60*
	**60-70**	45.9 [43.5-48.3]	47.8 [46.7-48.9]	46.2 [45.1-47.3]	45.9 [44.7-47.1]
	**Δ**	--	1.90	0.27	-0.02
	**70+**	47.2 [45.7-48.7]	45.4 [44.1-46.7]	44.8 [43.5-46.1]	43.8 [42.4-45.1]
	**Δ**	---	-1.84 (p=0.05)	-2.40 (p=0.015)	-3.47*
**Mental**	**Overall**	54.6 [53.7-55.4]	55.1 [54.4-55.8]	54.5 [53.8-55.2]	54.5 [53.7-55.3]
	**Δ**	---	0.53	-0.02	-0.06
	**60-70**	54.4 [53.0-55.8]	55.0 [54.1-55.9]	55.2 [54.3-56.0]	54.8 [53.8-55.8]
	**Δ**	---	0.56	0.79	0.38
	**70+**	54.5 [53.4-55.6]	55.2 [54.2-56.2]	53.5 [52.3-54.6]	54.4 [53.2-55.6]
	**Δ**	---	0.70	-1.06	-0.14
**Physical Activity Scale for Elderly**	**Overall**	127.2 [121.2-133.2]	125.2 [119.8-130.6]	127.8 [121.5-134.1]	110.7 [104.7-116.6]
	**Δ**	---	-2.04	0.55	-16.6*
	**60-70**	132.6 [124.0-141.2]	136.7 [129.2-144.2]	136.3 [127.6-144.9]	122.5 [114.7-130.3]
	**Δ**	---	3.09	3.67	-10.1
	**70+**	116.4 [108.9-124.0]	108.6 [101.1-116.2]	113.6 [105.2-122.1]	93.2 [84.2-102.2]
	**Δ**	---	-7.80	-2.80	-23.2*
**Late-Life Disability Index**					
**Frequency**	**Overall**	55.5 [54.8-56.1]	55.1 [54.5-55.6]	55.3 [54.7-55.8]	55.1 [54.4-55.7]
	**Δ**	---	-0.44	-0.25	-0.46
	**60-70**	56.0 [55.1-56.9]	55.6 [54.8-56.4]	55.7 [54.9-56.5]	55.6 [54.8-56.4]
	**Δ**	---			
	**70+**	54.7 [53.8-55.7]	54.3 [53.5-55.5]	54.5 [53.6-55.5]	54.2 [53.2-55.2]
	**Δ**	--	-0.40	-0.28	-0.40
**Limitation**	**Overall**	82.2 [80.6-83.8]	82.3 [80.8-83.6]	79.6 [78.2-81.0] (p=0.02)	78.8 [77.3-80.3] (p=0.002)
	**Δ**	---	0.05	-2.60	-3.4
	**60-70**	83.0 [80.5-85.4]	83.9 [82.0-85.8]	81.0 [79.2-82.9]	80.5 [78.7-82.3]
	**Δ**	---	0.97	-1.91	-2.42
	**70+**	81.3 [78.9-83.6]	79.6 [77.4-81.7]	77.6 [75.4-79.8]	76.1 [73.5-78.7]
	**Δ**	---	-1.70	-3.68 (p=0.02)	-5.16 (p=0.03)
**Gait Speed**	**Overall**	1.25 [1.22-1.29]	1.24 [1.22-1.26]	1.23 [1.22-1.25]	1.19 [1.17-1.21]
	**Δ**	---	0.02	-0.02	-0.07*
	**60-70**	1.25 [1.18-1.33]	1.27 [1.25-1.30]	1.26 [1.24-1.28]	1.23 [1.21-1.26]
	**Δ**	---	0.02	0.04	-0.02
	**70+**	1.23 [1.20-1.26]	1.18 [1.15-1.21]	1.19 [1.16-1.22]	1.12 [1.08-1.15]
	**Δ**	---	-0.05 (p=0.019)	-0.04 (p=0.05)	-0.11*

Values represent mean score (95% confidence interval) of the indicated metric adjusted for: Age, sex, education level, race, Charlson co-morbidity index, smoking status, knee OA, hip pain and cohort type (incident, progression or control), and baseline scoring (SF-12, PASE) where available. Values in 25-50%, 50-75%, high WC quartile columns represent difference from referent category (low WC). Models stratified by age adjust for the similar co-variates other than age.

**Table 6:** **Multivariable Regression Analysis of Follow-up Primary Outcome Measures (n=2182) – Using Charlson Co-Morbidity Index as a Co-Variate**

		Waist	Circumference	Quartile	
		Low WC	25-50% WC	50-75% WC	75%+
**Short-Form 12 Score**					
**Overall Score**	**OA +**	97.3 [93.4-101.2]	100.9 [99.5-102.2]	99.1 [97.8-100.4]	98.9 [97.6-100.2]
	**Δ**	---	3.53	1.74	1.60
	**OA -**	104.1 [102.7-105.4]	103.3 [101.7-104.8]	102.7 [101.1-104.3]	101.7 [99.8-103.6]
	**Δ**	---	-0.78	-1.35	-2.4 (p=0.03)
**Physical**	**OA +**	45.4 [43.6-47.2]	45.6 [44.5-46.6]	44.1 [43.0-45.2]	44.0 [42.9-45.0]
	**Δ**	---	-0.29	-0.85	-1.74
	**OA -**	49.2 [48.0]	48.9 [47.6-50.2]	48.3 [46.9-49.7]	47.4 [46.0-48.9]
	**Δ**	--	0.16	-1.32	-1.41
**Mental**	**OA +**	54.1 [52.7-55.4]	55.3 [54.3-56.2]	54.8 [53.9-55.8]	54.9 [53.9-55.8]
	**Δ**	---	1.19	0.77	0.80
	**OA -**	54.9 [54.0-55.9]	54.5 [53.6-55.5]	54.2 [53.1-55.2]	53.8 [52.5-55.1]
	**Δ**	----	-0.39	-0.77	-1.15
**Physical Activity Scale for Elderly**	**OA +**	124.7 [116.6-132.8]	120.7 [114.4-127.0]	126.7 [119.0-132.3]	109.2 [101.9-116.5]
	**Δ**	--	-3.95	1.99	-15.5 (p=0.004)
	**OA -**	132.0 [126.6-140.4]	133.4 [123.4-143.5]	128.3 [117.1-139.4]	112.7 [102.9-122.5]
	**Δ**	--	1.42	-3.74	-19.3 (p=0.003)
**Late-Life Disability Index**					
**Frequency**	**OA +**	55.1 [54.2-56.0]	55.0 [54.3-55.7]	54.7 [54.1-55.4]	54.6 [53.9-55.4]
	**Δ**	--	-0.10	-0.38	-0.47
	**OA -**	56.2 [55.4-57.1]	55.0 [54.1-55.9]	56.3 [55.1-57.4]	55.8 [54.6-57.0]
	**Δ**	--	-1.22 (p=0.05)	0.02	-0.41
**Limitation**	**OA +**	80.7 [78.4-83.0]	81.3 [79.5-83.1]	79.3 [77.6-81.0]	78.2 [76.4-79.9]
	**Δ**	---	0.60	-1.38	-2.55
	**OA -**	84.0 [82.0-86.0]	83.9 [81.5-86.2]	80.5 [77.8-83.2]	79.9 [77.1-82.7]
	**Δ**	---	-0.12	-3.49 (p=0.04)	-4.10 (p=0.02)
**Gait Speed**	**OA +**	1.23 [1.17-1.29]	1.22 [1.19-1.24]	1.22 [1.20-1.24]	1.17 [1.15-1.19]
	**Δ**		-0.02	-0.01	-0.06 (p=0.046)
	**OA -**	1.29 [1.27-1.31]	1.27 [1.24-1.30]	1.26 [1.23-1.29	1.21 [1.18-1.24]
	**Δ**	---	-0.02	-0.03	-0.08*

**Δ** – represents the adjusted difference between each tertile of Waist Circumference (WC) and low WC. Models were adjusted for age, sex, race, education, Charlson co-morbidity index, smoking status, and cohort type. Baseline scores were included where available.

**Table 7:** **R** ^**2**^ **of Multivariable waist circumference or body mass index modeling**

		R ^2^ of model with	Charlson index	R ^2^ of model with	Modified charlson index
		Waist circumference	Body mass index	Waist circumference	Body mass index
**Short-Form 12 Score**					
**Overall Score**	**Overall**	0.4011	0.4054	0.3879	0.3943
	**60-70**	0.407	0.3894	0.3876	0.3971
	**70+**	0.41352	0.902	0.3902	0.3931
**Physical**	**Overall**	0.3147	0.3187	0.3171	0.3194
	**60-70**	0.3154	0.3210	0.3104	0.3146
	**70+**	0.3102	0.3093	0.3274	0.3236
**Mental**	**Overall**	0.2308	0.2313	0.2065	0.2094
	**60-70**	0.225	0.2258	0.2089	0.2126
	**70+**	0.2591	0.2564	0.2233	0.2226
**Physical Activity Scale for**	**Overall**	0.2443	0.2425	0.2606	0.2576
**Elderly**	**60-70**	0.2132	0.2019	0.2298	0.2306
	**70+**	0.2284	0.2181	0.2550	0.2444
**Late-Life Disability Index**					
**Frequency**	**Overall**	0.1022	0.1022	0.0866	0.0881
	**60-70**	0.0967	0.0969	0.0849	0.0881
	**70+**	0.1006	0.1007	0.0709	0.0962
**Limitation**	**Overall**	0.0936	0.0916	0.0912	0.913
	**60-70**	0.0987	0.0983	0.101	0.107
	**70+**	0.0754	0.0711	0.0873	0.0820
**Gait Speed**	**Overall**	0.5377	0.05342	0.5277	0.5226
	**60-70**	0.5019	0.4998	0.4921	0.4911
	**70+**	0.5469	0.5382	0.5528	0.5368

All models adjust for age, sex, education level, race, smoking status, knee OA, hip pain and cohort type (incident, progression or control), and baseline scoring (SF-12, PASE) where available. Standard Charlson Co-morbidity Index is accounted for in the R^2^ in the first two columns, while a modified Charlson Co-morbidity index accounting for Chronic Obstructive Pulmonary Disease, Peptic Ulcer Disease, Kidney Disease, Kidney Transplant, Rheumatoid Arthritis and Polymyalgia Rheumatica.

## Results

Statistical differences were observed in age, although there was a higher proportion of females in the low WC quartile (Table 1). Charlson co-morbidity score and its modified version, WOMAC scores, and number of medications increased with increasing WC quartile. The proportion of subjects with knee OA increased by quartile. Comparing subjects included in our analysis to those excluded demonstrated that those excluded (either having a knee arthroplasty or have died) were older, less likely to be female, and have higher comorbidity scores and number of medications. They had lower SF-12 scores at baseline but no differences in PASE scores [Data not shown]. Notably there was high correlation between body mass index and waist circumference (r = 0.79).

The unadjusted functional outcome data are presented in Table 2. Outcomes are compared between WC groups at baseline and six years and outcomes are compared across time in each group. Overall and physical component scales of the SF-12 dropped with increasing waist circumference both at baseline and follow-up, and dropped significantly within each category between baseline and follow-up. Mental component scores did not change. There were marked parallel declines in PASE scores over time and between groups at each baseline and 6 years follow-up. LLDI scores diminished at follow-up and the proportion of ADL impairments increased significantly from 18.0% in the lowest WC quartile to 36.6% in the upper WC quartile.

Our multivariable regression models are presented in Table 3 (overall scores and stratified by age) and Table 4 (stratified by OA status). In examining by WC quartile, SF-12 decline appears to be age-dependent and subjects in the high WC quartile had markedly lower scores than the lowest quartile. High WC quartile subjects had significant declines in PASE scores as compared to the other categories but only in the ≥70 year age group. The limitation component of the LLDI was most impacted in the ≥70 year age group as compared to the low WC quartile. Lastly, gait speed was lowest in the highest WC quartile as compared to the other categories. Generally, the adjusted estimates were similar adjusting for either the Charlson score or the modified Charlson co-morbidity index (Table 5). Patients with osteoarthritis generally had lower outcome measures as compared to those without OA (Table 6). However, significant differences were observed in PASE scores in both cohorts, yet only those without OA had differences compared to the low WC quartile for the limitation domain of the LLDI and gait speed. Model R^2^ were similar for predictor variables for both WC and BMI (Table 7).

## Discussion

Older adults with high waist circumference are at risk for reduced self-reported health, physical activity scores, and disability. Our results also suggest that the presence of OA leads to markedly reduced quality of life and physical activity scores than in subjects without OA in those with elevated visceral adiposity.

Elevated waist circumference has been associated with progression of OA in both cross-sectional and longitudinal epidemiological studies [25, 26]. Our study specifically targets the association of WC with geriatric outcomes, including disability, quality of life and physical activity that have not been adequately evaluated longitudinally. Previous findings have demonstrated inconsistent relationships between WC and functional decline in older adults [27, 28]. Yet, our results are consistent with those [29] who demonstrated that the highest WC quartile was associated with mobility and agility disability in an older cohort at 2-year follow-up. We found robust relationships between visceral obesity with respect to degree of functional impairment elderly persons over time suggesting a compelling rationale for clinician encouragement of weight reduction targeting visceral or central obesity in older adults at risk for osteoarthritis.

We found that after stratification by OA status, physical component scores of the SF-12, PASE score, and LLDI scores were consistently lower in those with OA. Additionally, gait speed was lower in this cohort. These results are not surprising but the differences between high WC quartile and lowest appeared to be present only in those without OA. These findings suggest that the rate of decline between WC quartiles likely has plateaued well before the development of OA. While purely hypothesis generating, this data could suggest the importance of identifying visceral adiposity in a population at risk knee OA population. Providers should consider promote weight loss to delay physical disability in this population.

Our findings underscore the importance of visceral adiposity measured by easily obtained clinical variables as an important and easily obtained metric with significant predictive value in long-term outcomes for older adults at risk for osteoarthritis. Yet caution is warranted in interpreting our results that may underestimate the natural history of decline observed. First, our cohort was relatively young (mean was ~68 years). The onset of disability increases with increasing age [1] and hence longer follow-up may be needed to delineate the natural course of the obesity in relation to OA. Frailty and disability often are preceded by years of preserved or compensated functional decline which would not be reflected in our findings. Second, the recruitment was based on a community-based cohort who would be able to participate in this clinical trial and may not be fully representative of the general population of older adults in the community. Third, the degree of co-morbidity was quite modest in the study population, implying a healthier population. While these results provide a reasonable basis for future study of important longitudinal geriatric outcomes, the magnitude of our changes are modest and may not necessarily be clinically significant. A longer follow-up with a cohort with advanced medical and sociodemographic characteristics would be required to confirm our results in a more medically and functionally compromised population of older adults. Lastly, WC quartiles as our main predictor was in line with previous author’s recommendations that it facilitates comparison of anthropometric indices between studies [23]. Incorporating WC as a continuous variable would require polynomial regression modeling, which would lead to challenges with interpretability of our results from a clinical standpoint.

We acknowledge a number of other limitations in our study. While the dataset was designed to observe longitudinal outcomes specific to OA, our analysis may not have coincided with the primary scope of the initial study design. Additionally, of those aged 60 or greater, we were missing data on a number of distal (six-year) outcomes. While we attempted to mitigate this issue by comparing included subjects vs. non-included subjects, and used a statistical methodology to account for such issues, our missing cohort appeared to have higher degree of comorbidity and socioeconomic status, suggesting that our results may indeed be an underestimate of the true effect observed. Importantly, our analysis differed from one published by Colbert et al. [30], who utilized the OAI and its four-year outcomes to assess the impact of obesity and race on gait speed and WOMAC scores [31]. Although our data was consistent with theirs, these authors did not specifically look at older adults, nor did they utilize PASE, SF-12 or LLDI as primary outcomes. Our results are at risk for possible over-adjustment as well. We deliberately presented unadjusted data to demonstrate the similar trends after accounting for *a priori* variables that we believed could impact our estimates. We agree that certain variables including social support, depression, and physical measures could conceivably have been omitted and/or excluded in our analysis. To account for possible confounding between predictor variables in the Charlson index, we restricted our modified index to those factors likely not influenced by obesity or disability outcomes. Although the estimates were slightly different, the trends were not. Lastly, future studies should relate anthropometric with body composition measures and distal outcomes.

BMI fails to differentiate between central and peripheral fat stores [8]. These results focus solely on the impact of central adiposity on functional outcomes in older adults. We previously demonstrated in a similar population that BMI impacts quality of life and physical function [Batsis et al. Under Review, Public Health Nutrition], and this current study suggests that abdominal obesity could be a separate predictor of poor functional outcomes. Our intent in this study was not to differentiate which anthropometric measures is superior in predicting distal outcomes. We were unable to adjust for BMI in our modeling as the two variables were highly collinear. This was not surprising in this sample as study recruitment focused on those with risk factors for OA (including overweight and obese based on BMI) making the metrics more homogenous. We believe that future studies should consider not only the impact of elevated WC in normal BMI individuals, but the corollary to identify the potential contribution of each metric. Particularly, in older adults, there is a strong consensus that other simple anthropometric measures should be considered for assessment of adiposity, over and above BMI. While the R^2^ in our modeling did not differ greatly (Table 7), using WC may still be an important anthropometric variable to measure. Not considering WC as an alternative measure may ignore a considerable sample of subjects at otherwise risk of adverse outcomes as we previously have demonstrated [32] suggesting the need for future analyses to determine the impact of these combined metrics on outcomes.

Lastly, sarcopenia, the loss of muscle mass and quality with aging [33, 34], impacts function and quality of life, and may be particularly present in subjects with OA [35]. WC is strongly associated with sarcopenic obesity and insulin resistance [36], both of which are related to functional decline, frailty and disability, and thought to be partially mediated by an increase in pro-inflammatory markers, including IL-1, IL-6, and TNF-a. Additionally, leptin levels are also associated both prevalent and incident central obesity and OA [37]. We suspect that there may be a link between the functional decline observed in the highest WC group in those with OA that may potentially be leptin-mediated. We also suspect that higher degrees of pro-inflammatory cytokines viscerally may be implicated in this phenomenon. Whether the alterations in body composition due to progressive replacement of muscle mass by fat mass, in addition to loss of skeletal muscle mass and function in the aging process may be implicated in this functional decline needs further evaluation. Future study should further examine whether leptin and changes in pro-inflammatory cytokine levels predict functional impairment longitudinally.

## Conclusion

In older adults, high waist circumference is associated with worsening self-reported quality of life and physical activity scores. Clinicians should consider not only targeting this subgroup for aggressive weight management to prevent such functional decline, but also encourage strengthening exercises and increases in physical activity levels which both can improve joint pain and may improve overall walking performance.
